# Transcatheter edge‐to‐edge repair of atrial secondary mitral regurgitation positively influences atrial remodelling

**DOI:** 10.1002/ehf2.15252

**Published:** 2025-03-11

**Authors:** Aniela Petrescu, Martin Geyer, Julian Andres Gelves Meza, Omar Hahad, Tobias Ruf, Valeria Maria de Luca, Lukas Hobohm, Theresa Gößler, Felix Kreidel, Philipp Lurz, Ralph Stephan von Bardeleben

**Affiliations:** ^1^ Department of Cardiology, Cardiology I University Medical Center Mainz of the Johannes Gutenberg‐University Mainz Mainz Germany; ^2^ Fundacion Cardioinfantil Instituto de Cardiologia Bogota Colombia; ^3^ Department of Medicine and Surgery Universita Campus Bio‐Medico di Roma Rome Italy; ^4^ Department of Cardiology Universitätsklinikum Schleswig‐Holstein, Campus Kiel Kiel Germany

**Keywords:** Atrial secondary mitral regurgitation, Echocardiography, Percutaneous edge‐to‐edge repair

## Abstract

**Background:**

Atrial secondary mitral valve regurgitation (ASMR) is a distinct anatomical subset of secondary mitral regurgitation (SMR). Evidence of the effect of transcatheter edge‐to‐edge repair (TEER) on left atrial (LA) anatomy and function, especially reverse remodelling (LARR), is still sparse.

**Methods and results:**

We retrospectively evaluated all consecutive patients treated with TEER for mitral regurgitation (MR) in our centre between January 2013 and October 2023. Of the 597 patients with SMR, 103 patients (17.3%) met the inclusion criteria for ASMR. All patients in the ASMR group (mean age 79.4 ± 6.8 years, 71% female) were symptomatic (89% NYHA ≥ III) and had a mean logistic EuroScore of 22.5 ± 12.4%. TEER was successfully performed in all patients, and invasive LA mean pressures decreased intraprocedurally from 17.8 ± 5.7 to 13.1 ± 4.8 mmHg (*P* < 0.001). At hospital discharge, 94% of patients had mild residual or non/trace MR. At 1YFUP, the prevalence of residual moderate MR was 7% and 1% had severe MR. A significant reduction in LA volume compared with baseline, both at end‐systole (151.4 ± 64 vs. 113 ± 64 mL, *P* < 0.001) and at end‐diastole (119.8 ± 56 vs. 91.2 ± 56.9 mL, *P* < 0.001) could be observed. Seventy per cent of patients had a sustained decrease in NYHA class ≤ II. LARR, defined as LAESV decrease ≥15% at 1YFUP, was documented in 59% of patients. These patients were more likely to have lower post‐interventional mitral valve mean pressure gradients (2.2 ± 0.8 mmHg vs. 2.8 ± 1.1 mmHg, *P* = 0.02) and lower BNP at discharge and at 1 month follow‐up [319 (197.8 to 526) vs. 560 (279.3 to 929), *P* = 0.07, and 287.5 (191.3 to 386.3) vs. 506.5 (223.3 to 935.5), *P* = 0.06, respectively]. A multivariate logistic regression analysis identified pre‐procedural MPG (*P* = 0.06, OR 0.92, CI 95% 0.85–1.00) and BNP at discharge (*P* = 0.11, OR 0.99, CI 95% 0.99–1.00) as independent predictors for the occurrence of LARR at 1 year.

**Conclusions:**

Transcatheter mitral valve repair by edge‐to‐edge therapy represents a safe and effective therapeutic option in symptomatic patients with atrial secondary mitral regurgitation and might have the potential to induce left atrial reverse remodelling.

## Introduction

Secondary mitral regurgitation (SMR) is the result of an insufficient coaptation of the mitral valve (MV) leaflets due to pathologies of the surrounding tissues, while the valve apparatus is lacking relevant damage.^1^ In most patients, this is caused by left ventricular (LV) systolic dysfunction and remodelling. However, in a smaller subset of patients, SMR is solely related to left atrial (LA) dilatation in the absence of a ventricular pathology. In those, the term of ‘atrial secondary’ MR (ASMR) has been established to discriminate from the more common ventricular form of SMR.^2^ ASMR occurs commonly in the presence of atrial fibrillation (AFib) or heart failure with preserved ejection fraction (HFpEF).^3^ These underlying conditions lead to the so called ‘atrial remodelling’, including interstitial fibrosis with decreased LA elastance, and increased LA pressures. This causes pathological dilatation of the LA as well as of the mitral annulus, which leads to mitral leaflet malcoaptation.^4^ Timely intervention of ASMR may arrest the ‘vicious circle’ of LA remodelling with consequent reduction in LA size and improved function.^5^

Despite the fact that different SMR entities have various pathophysiological backgrounds and clinical courses, current guidelines still do not address them separately.^6^ ASMR is amenable to current surgical and interventional repair techniques,^7^, ^8^, ^9^, ^10^, ^11^, ^12^ yet the long‐term effect of transcatheter mitral valve repair by edge‐to‐edge‐repair (M‐TEER) on LA enlargement in this subpopulation is poorly studied.

The purpose of the present study was to evaluate the baseline clinical and echocardiographic characteristics of patients with ASMR, as well as to explore the potential of atrial reverse remodelling after M‐TEER and its impact on the clinical outcomes at long‐term follow‐up.

## Methods

### Study population

All consecutive patients undergoing M‐TEER in our university centre between January 2013 and October 2023 were analysed in a retrospective monocentric study. All patients had high‐grade MR despite maximal tolerated guideline directed therapy. Decision for timing and choice of treatment (surgical vs. interventional) were made by an interdisciplinary heart team based on anatomy and individual risk factors including risk scores (e.g., logistic EuroSCORE^13^), frailty and co‐morbidities.

### Echocardiographic analysis

All echocardiograms were retrospectively analysed by an experienced operator (AP). The mechanism of MR was evaluated by transthoracic (TTE) and transoesophageal echocardiography (TEE). MR severity was denominated in three grades: ‘mild’, ‘moderate’ and ‘severe’ and was assessed applying an integrative approach of quantitative, semiquantitative and qualitative parameters according to current guidelines^14^, ^15^.^16^ The anteroposterior and intercommissural annular dimensions were measured on the long‐axis and intercommissural TEE MV views. The assessment of tricuspid regurgitation (TR) severity was performed according to society recommendations, with the ‘severe’ grade comprising the recently proposed subgrades ‘severe’, ‘massive’ and ‘torrential’.^15^, ^17^ Cardiac chambers' size and function were quantified according to the European Society of Cardiology recommendations.^18^ LV and LA volumes and function were evaluated using the Simpson's biplane method of disks and provided indexed to body‐surface area (as assessed according to Mosteller formula). The right ventricular systolic pressure was estimated based on the TR jet maximum velocity and estimated right atrial pressure. The ultrasound machines used were Philips iE33 and Epiq 7C (Philips, Andover, MA, USA) and GE Vivid E95 (GE Healthcare, Chicago, IL, USA). The analysis was conducted using Intellispace Cardiovascular and QLAB (Philips). Measurements were performed at baseline (pre‐procedural) and discharge and at 1 year follow‐up.

### Procedural technique

M‐TEER was performed using MitraClip®‐systems (Abbott Structural Heart, Santa Clara, California, USA) as previously described.^19^, ^20^ In 15 patients, M‐TEER was performed using a PASCAL system (Edwards Lifesciences, Irvine, California). LA pressures were invasively measured peri‐interventionally before and after device implantation.

### Definitions

ASMR was defined as SMR with (1) relevant LA dilatation (LAVi > 34 mL/m^2^ or LA diameter > 40 mm), and (2) no significant LV systolic dysfunction (ejection fraction, EF > 45%) or dilatation (LV enddiastolic volume index, LVEDVi ≤ 89 mL/m^2^ for male and ≤70 mL/m^2^ for female).^8^, ^9^, ^21^, ^22^

Left atrial reverse remodelling (LARR) was defined, as in most reports, as the change in LA end‐systolic volume (LAESV) of ≥15% from baseline to 1‐year follow‐up (1YFUP), as assessed with TTE using biplane Simpson's method.^23^, ^24^ The change in LAESV was calculated as follows: (LAESV at baseline − LAESV at 1YFUP)/LAESV at baseline × 100. Based on this definition, the study population was further stratified accordingly into patients with LARR vs. those without (NLARR). Clinical characteristics, procedural and long‐term outcomes were compared between the abovementioned subgroups.

According to the recommendations of the Mitral Valve Academic Research Consortium (MVARC), technical success was defined as successful access, delivery and retrieval of the device delivery system and successful device deployment without procedural death or emergent surgery or reintervention. Due to the retrospective study design, procedural success was adjusted to discharge conditions and was defined as reduction of MR to ‘acceptable’ levels without significant MV stenosis (MR ≤ 2, mean transvalvular pressure gradient (MPG) < 5 mmHg) achieved after successful placement of the device in the absence of major peri‐procedural complications.^25^

### Follow‐up

Clinical follow‐up was obtained at discharge and at 1 year by clinical visits. Among clinical examinations, symptoms were primarily evaluated by the New York Hear Association (NYHA) functional class. Other collected key outcome measures included MR severity, presence of LARR, change of brain natriuretic peptide (BNP) and survival status.

### Statistical analysis

Continuous variables are presented as means ± standard deviations or median with interquartile range (IQR) when appropriate. Normality of the continuous data was checked using the Kolmogorov–Smirnov test. Categorical variables were reported as absolute and relative frequencies. Statistical comparisons for categorical variables were made by Fishers exact or *χ* ^2^ tests. For continuous variables, Student's *t* tests, Wilcoxon rank sum tests or Mann–Whitney *U* test were used, as appropriate. To identify predictors of LARR, a multivariate logistic regression analysis using a backward stepwise algorithm was applied for the cohort of 51 patients with available 1YUP to all potential influential baseline parameters from the univariate analysis (*P* < 0.10) and to those considered clinically important in order to calculate independent predictors of LARR. Each result was reported as odds ratio (OR) and corresponding 95% CI.

All tests were two‐sided and *P* values <0.05 were considered as statistically significant. Statistical analysis was performed with SPSS (IBM SPSS Statistics for Windows, Version 29.0.1.0).

## Results

### Patient characteristics

**Figure 1:**
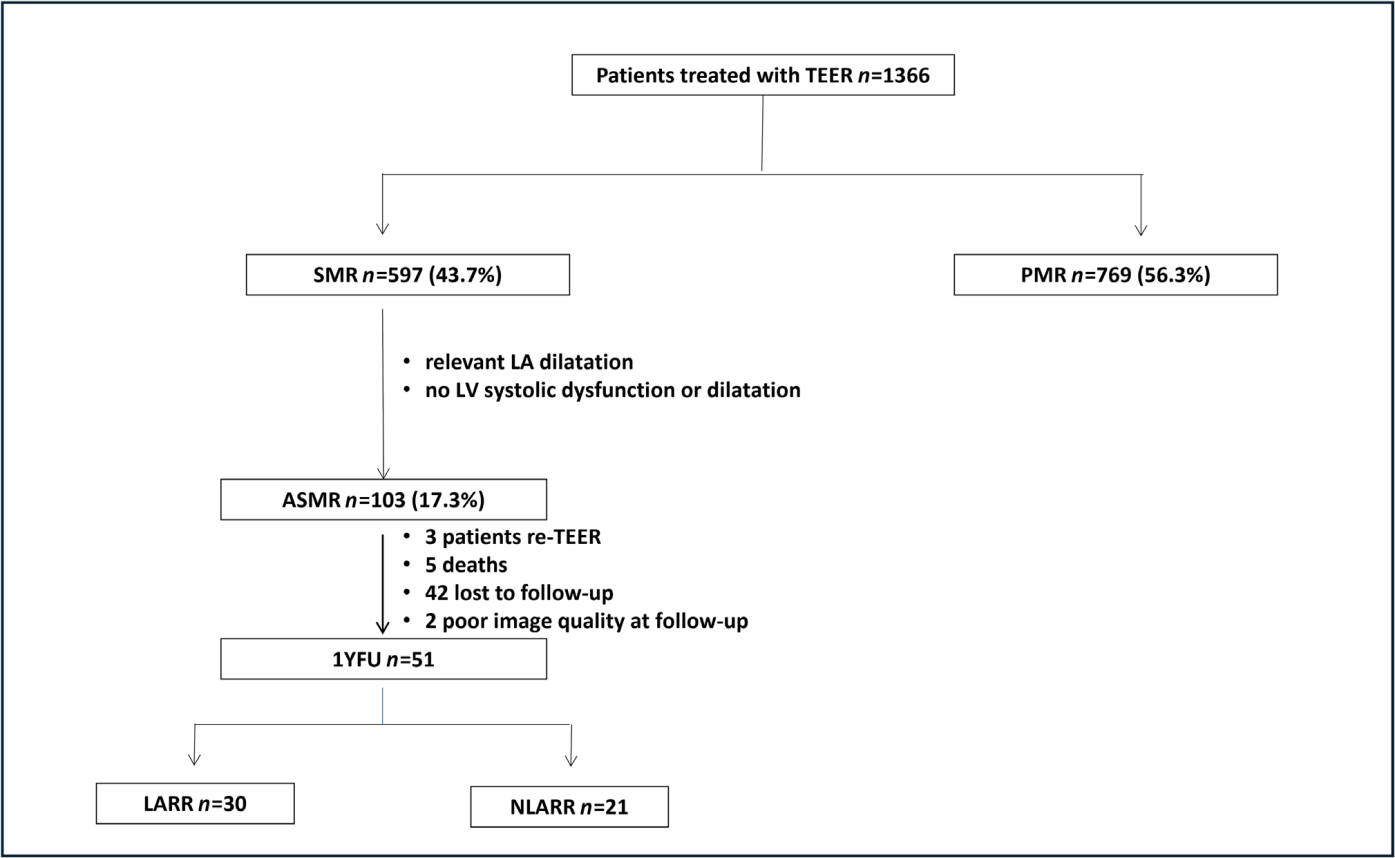
Flow diagram showing the selection process of ASMR cohort: A total of 1366 patients consecutively treated with edge‐to‐edge transcatheter mitral valve repair (M‐TEER) were screened. Among these, 597 patients had secondary mitral regurgitation (SMR), and 769 patients had primary mitral regurgitation (PMR). After the application of inclusion criteria, 103 patients were included in the atrial functional mitral regurgitation (ASMR) group. Paired clinical and echocardiographic data was available in 51 patients at 1 year follow‐up (1YFUP). According to the presence of left atrial reverse remodelling (LARR) at 1YFUP, further stratification was done in patients with LARR vs. patients without (NLARR). LA, left atrial.

We retrospectively screened all 1366 patients treated with M‐TEER in our centre between January 2013 and October 2023. Of the 597 patients (43.7%) with severe SMR, 103 patients (17.3%) had ASMR according to the pre‐specified criteria and were included in the final analysis (*Figure* *1*). Baseline patient characteristics including clinical data and echocardiography are reported in *Tables* *1* and *2*. All patients (mean age 79.4 ± 6.8 years, 71% female) were symptomatic and were at high or prohibitive risk for cardiac surgery, with a logistic EuroScore of 22.5 ± 12.4%. The majority suffered from heart failure of NYHA functional class ≥ III (89%). A history of atrial fibrillation (AFib) was documented in 92 patients (89%) of whom 73 patients (79%) had either persistent or permanent AFib. The prevalence of arterial hypertension was 84%. Most patients were taking beta‐blockers (80%), angiotensin‐converting enzyme inhibitor or angiotensin II receptor blocker (75%), and/or loop diuretics (86%). Baseline echocardiography showed increased LA volumes [left atrial end‐diastolic volume (LAEDV, 119.8 ± 56.1 mL), LAESV (151.44 ± 64 mL) and LAVi (103 ± 42.3 mL/m^2^)].

**Table 1:** Baseline clinical characteristics of ASMR patients undergoing M‐TEER

Age (years)	79.4 ± 6.8
Height (m)	1.7 ± 8.6
Weight (kg)	75 ± 19.4
BSA	1.8 ± 0.5
Sex (male), *n* (%)	30 (29)
Logistic EuroScore	22.5 ± 12.4
eGFR (mL/min)	48.3 ± 20.6
NYHA functional class, *n* (%)	
NYHA I, *n* (%)	0 (0)
NYHA II, *n* (%)	13 (13)
NYHA III, *n* (%)	81 (79)
NYHA IV, *n* (%)	8 (8)
Diabetes, *n* (%)	12 (12)
Hypertension, *n* (%)	87 (84)
Previous myocardial infarction, *n* (%)	19 (18)
Previous PCI, *n* (%)	22 (21)
Previous CABG, *n* (%)	12 (12)
Previous stroke, *n* (%)	17 (17)
COPD, *n* (%)	14 (14)
AFib, *n* (%)	92 (89)
Persistent, *n* (%)	34 (33)
Permanent, *n* (%)	39 (38)
Previous ICD, *n* (%)	1 (1)
Previous CRT, *n* (%)	4 (4)
BNP, pg/L	372.0 (208, 685.5)
Medication, *n* (%)	
Loop diuretics	89 (86)
ACEI or ARB	77 (75)
Beta‐blockers	82 (80)
Aldosterone antagonists	31 (30)

Data are presented as *n* (%), mean (SD) or median (interquartile range).

ACEI, angiotensin‐converting enzyme inhibitor; AFib, atrial fibrillation/flutter; ARB, angiotensin receptor blocker; BSA, body surface area; CABG, coronary artery bypass graft; COPD, chronic obstructive pulmonary disease; CRT, cardiac resynchronization therapy; MR, mitral regurgitation; NYHA, New York Heart Association; PCI, percutaneous coronary intervention; ICD, implantable cardioverter defibrillator.

**Table 2:** Baseline echocardiographic parameters of ASMR patients undergoing M‐TEER

LVEF biplane (%)	57 ± 8
LVEDVi (mL/m^2^)	57 ± 21.4
LVESVi (mL/m^2^)	24.5 ± 11.6
LAV (mL)	180 ± 77.1
LAVI (mL/m^2^)	103 ± 42.3
LA‐EF biplane (%)	21.3 ± 10.5
LA_A_ (mm^2^)	36 ± 9.7
LA_L_ (mm)	7.4 ± 1.0
MR grade, *n* (%)	
2	12 (12)
3	91 (88)
MR VC (biplane, mm)	0.8 ± 0.2
EROA (cm^2^)	0.2 ± 0.1
MR PISA (cm^2^)	0.7 ± 0.2
MR RV (PISA) mL	39 ± 19
MV MPG (mmHg)	1.8 ± 0.9
MVA ALPM (mm)	41 ± 6
MVA AP (mm)	43 ± 6
TR grade (n %)	
0	5 (5)
1	20 (19)
2	19 (18)
3	56 (54)
TVmax (m/s)	3.1 ± 0.7
E (cm/s)	103 ± 28.7
E′ med (cm/s)	7.8 ± 3
E′ lat (cm/s)	11.5 ± 3.3
E/E′ average	12 ± 5.6

Data are presented as *n* (%), mean (SD) or median (interquartile range).

2CV, two chamber view; 3CV, three chamber view; 4CV, four chamber view; ALPM, anterolateral to posteromedial; AP, anteroposterior; E, early mitral inflow velocity; E/E′, ratio between early mitral inflow velocity and mitral annular early diastolic velocity; E′, mitral annular early diastolic velocity; EROA, effective regurgitant orifice area; LA_A_, left atrial area; LAEDV, left atrial end‐diastolic volume; LAEF, left atrial ejection fraction; LAESV, left atrial end‐systolic volume; LA_L_, left atrial length; LAV, left atrial volume; LAVI, left atrial volume index; LVEDV, left ventricular end‐diastolic volume; LVEF, left ventricular ejection fraction; LVESV, left ventricular end‐systolic volume; MR, mitral regurgitation; MV MPG, mitral valve mean pressure gradient; MVA, mitral valve annulus; PISA, proximal isovelocity surface area; RV, regurgitation volume; TR, tricuspid regurgitation; TVmax, tricuspid maximum velocity; VC, vena contracta.

### Peri‐interventional outcomes

**Figure 2:**
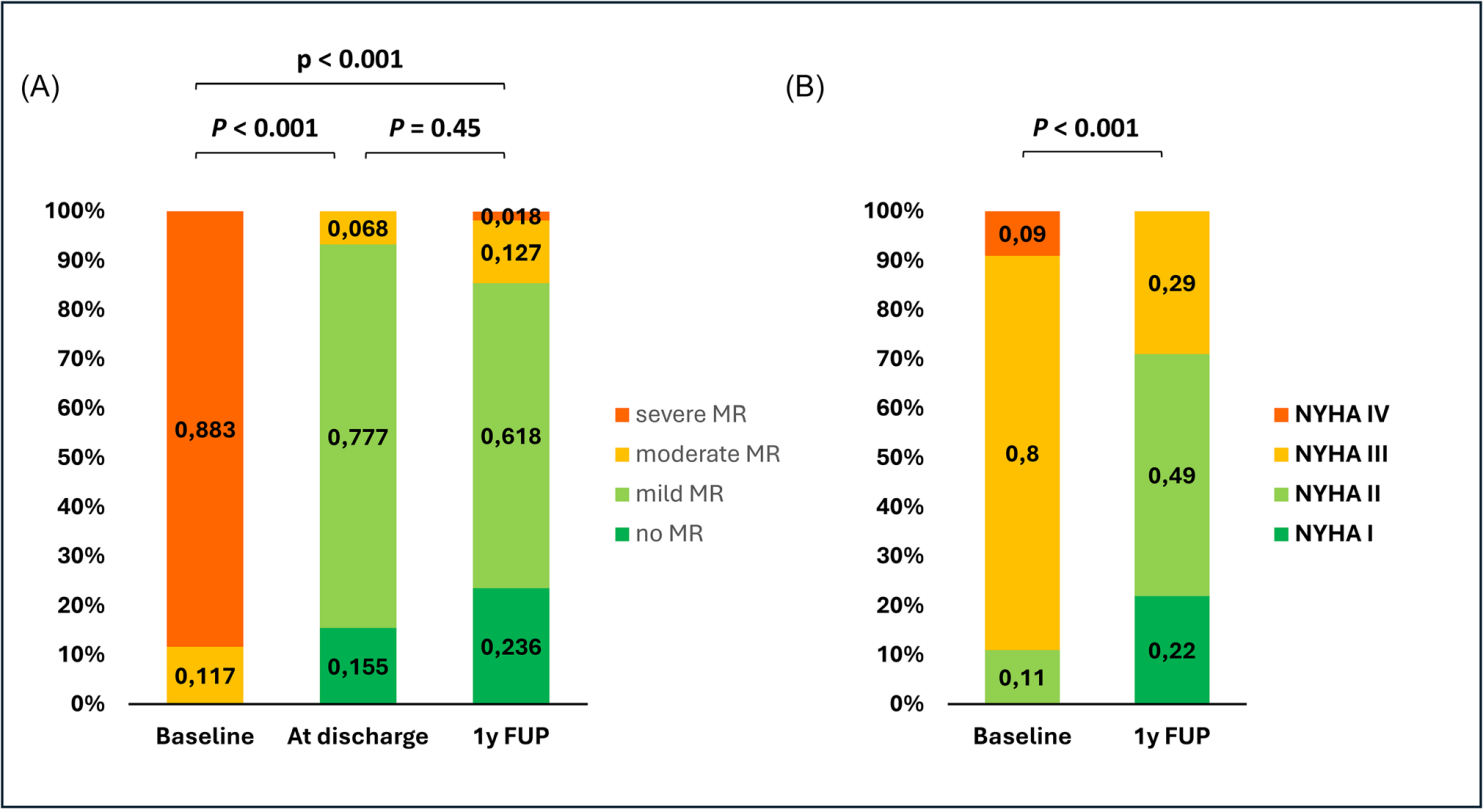
Clinical outcomes in patients with atrial functional mitral regurgitation: Mitral regurgitation (MR) severity (left) and New York Heart Association (NYHA) functional class (right) before and after transcatheter edge‐to‐edge repair among patients with atrial functional mitral regurgitation. The *p* values reflect comparisons of paired data.

Peri‐procedural data and echocardiographic outcomes are summarized in *Tables* *3* and *4*. M‐TEER was performed in all patients without any peri‐interventional major complications, with a technical success of 100% and a procedural success at discharge achieved in 92% of patients. Seven patients showed post‐procedural moderate residual MR (*Figure* *2* *A*). There was one hospital death caused by arrhythmia 4 days after the intervention.

**Table 3:** Peri‐procedural hemodynamic findings

	Pre‐procedural	Post‐procedural	*P* value
MR grade, *n* (%)			**<0.001**
0	0 (0)	16 (16)	
1	0 (0)	80 (78)	
2	12 (12)	7 (7)	
3	91 (88)	0 (0)	
MV MPG (mmHg)	1.8 ± 0.9	2.7 ± 1.3	**<0.0001**
LA a (mmHg)	19.8 ± 6.7	15.2 ± 5.9	**<0.0001**
LA v (mmHg)	28.4 ± 11.6	19.8 ± 8.8	**<0.0001**
LA m (mmHg)	17.8 ± 5.7	13.1 ± 4.8	**<0.0001**

Data are presented as *n* (%), mean (SD) or median (interquartile range).

1YFUP, 1‐year follow‐up; a, a‐wave; LA, left atrium; m, mean pressure; MPG, mean pressure gradient; MR, mitral regurgitation; v, v‐wave.

**Table 4:** Procedural data

Procedural success = 1, *n* (%)	95 (92)
Number of clips, *n* (%)	
1	78 (76)
2	24 (23)
3	1 (1)
Redo M‐TEER during 1YFUP, *n* (%)	3 (3)
Time to Redo M‐TEER (months)	5.1 ± 1.7
Concomitant tricuspid T‐TEER, *n* (%)	4 (4)
Tricuspidal T‐TEER during 1YFUP, *n* (%)	10 (10)
Time to T‐TEER (months)	4.5 ± 3.8

Data are presented as *n* (%) or mean (SD);

1YFUP, one‐year follow‐up; M‐TEER, mitral valve edge‐to‐edge therapy; T‐TEER, tricuspid valve edge‐to‐edge therapy.

Invasive LA pressure measurements were available in 65 patients (63%). In average, LA mean pressures decreased from 17.8 ± 5.7 mmHg to 13.1 ± 4.8 mmHg (*P* < 0.001). In 78 patients (76%), one device was implanted during index‐procedure, while 24 patients (23%) received two clips, and 1 patient receives three clips (1%). MV MPG increased post‐interventionally from 1.8 ± 0.9 mmHg to 2.7 ± 1.3 mmHg (*P* < 0.0001).

The rate of patients with severe TR at baseline was 54%. Of those, 25% underwent TEER of the tricuspid valve either concomitantly (7%, *n* = 4) during index‐visit or staged (in mean 4.5 ± 3.8 months post‐M‐TEER, 19%, *n* = 10).

A significant decrease in BNP values could be observed at discharge [369.5 (204.8 to 683.3) vs. 327.0 (208.8 to 545.8) *P* = 0.02].

### Outcomes at 1YFUP

Paired clinical and echocardiographic data were available in 51 patients (52% of the baseline cohort) over a median follow‐up time of 11 months (interquartile range: 11 to 13 months). Two patients had a recurrence of severe MR and underwent re‐TEER (5‐ and 7‐months post‐intervention, respectively), two patients had poor imaging of the LA, five deaths occurred during the follow‐up period due to arrhythmia (*n* = 1), right heart failure (*n* = 1), septic shock (*n* = 2) and cancer (*n* = 1), while the rest of patients was lost to follow‐up, mostly during COVID‐19 pandemic. Results are presented in *Table* *5*.

**Table 5:** Echocardiographic outcomes at 1‐year follow‐up

Baseline vs. 1YFUP	Reduction	*P* value
LAESV (mL)	−25.7 ± 34.8, CI −35.4 to −16.1	**<0.001**
LAEDV (mL)	−16.7 ± 29.6, CI −25 to −8.5	**<0.001**
LAV AL (mL)	−19.8 ± 45.46, CI −35.1 to −4.4	**0.01**
LAVI AL (mL/m^2^)	−12.8 ± 26.7, CI −21.8 to −3.8	**0.007**
LA_A_ (cm^2^)	−4.3 ± 5.6, CI −5.8 to −2.7	**<0.001**
LA_L_ (mm)	−0.3 ± 0.6, CI −0.5 to −0.1	**0.002**
MV MPG (mmHg)	1.7 ± 1.4, CI 1.3 to 2.1	**<0.001**

Data are presented as mean difference ± SD of mean difference.

LA_A_, left atrial area; LAEDV, left atrial end‐diastolic volume; LAESV, left atrial end‐systolic volume; LA_L_, left atrial length; LAV, left atrial volume; LAVI, left atrial volume index; MV MPG, mitral valve mean pressure gradient.

**Figure 3:**
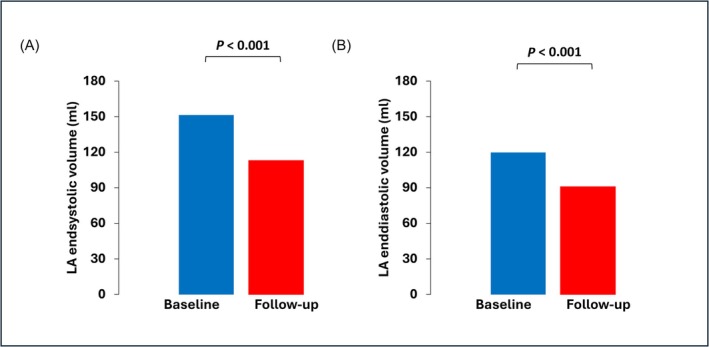
Left atrial reverse remodelling at 1 year follow‐up: Analysis at 1 year follow‐up showed a significant reduction in left atrial (LA) volume, both at end‐systole (128.8 ± 42.2 vs. 105.9 ± 47.9 mL, *P* < 0.001; panel A) and at end‐diastole (97.0 ± 36.1 vs. 80.3 ± 43.7 mL; *P* < 0.001; panel B).

In comparison with baseline measurements, a significant reduction in LA volume, both at end‐systole (151.4 ± 64 vs. 113 ± 64 mL, *P* < 0.001) and at end‐diastole (119.8 ± 56 vs. 91.2 ± 56.9 mL, *P* < 0.001; *Figure* *3*) could be documented at 1 year. An improvement in LAEF at 1 year was observed in 43% of patients with an increase from 19.1% ± 10.7% to 27.4% ± 10%. NYHA class I/II was found in 70% of patients (*Figure* *2* *B*). While 87% had no, trace or mild MR, the echocardiographic prevalence of residual moderate MR was 11%, and 2% of patients had severe MR at follow‐up (*Figure* *2* *A*).

### Characteristics according to left atrial reverse remodelling

LARR at 1 year was observed in 30 patients (59%). Clinical characteristics, procedural and long‐term outcomes were compared between the two subgroups (LARR vs. NLARR) and are depicted in *Table* *6*. While most relevant baseline characteristics including symptoms and proportion of residual MR exceeding moderate grade did not differ significantly between the groups, LARR‐patients showed a trend towards lower BNP at discharge and at 1 month follow‐up [319 (197.8 to 526) vs. 560 (279.3 to 929), *P* = 0.07, and 287.5 (191.3 to 386.3) vs. 506.5 (223.3 to 935.5), *P* = 0.06, respectively]. With regard to echocardiographic parameters, the LARR‐group showed significantly lower post‐procedural MPG (2.2 ± 0.8 mmHg vs. 2.8 ± 1.1 mmHg, *P* = 0.02), and numerically higher LA dimensions and better LA‐EF at baseline, however, without reaching a significant level. All other assessed echocardiographic parameters at baseline did not differ significantly between the two groups. A multivariate logistic regression analysis identified pre‐procedural MPG (*P* = 0.06, OR 0.92, CI 95% 0.85–1.00) and BNP at discharge (*P* = 0.11, OR 0.99, CI 95% 0.99–1.00) as independent predictors for the occurrence of LARR at 1 year.

**Table 6:** Characteristics of patients according to the left atrial reverse remodelling at 1 year follow‐up

	RR (*n* = 30)	NRR (*n* = 21)	*P* value
Clinical characteristics			
Age (years)	79.2 ± 6.8	78.9 ± 7.65	0.88
Height (m)	1.67 ± 0.08	1.63 ± 0.07	0.11
Weight (kg)	75.9 ± 16.6	71.6 ± 20.5	0.42
BSA (m^2^)	1.77 ± 0.4	1.64 ± 0.5	0.32
Sex (male), *n* (%)	9 (30)	4 (19)	0.52
Logistic EuroScore	20.5 ± 9.4	22.9 ± 14.3	0.48
eGFR (mL/min)	48.0 ± 20.5	44.7 ± 16.8	0.36
NYHA functional class, *n*, %)			0.91
NYHA I, *n* (%)	0 (0)	0 (0)	
NYHA II, *n* (%)	3 (10)	2 (10)	
NYHA III, *n* (%)	25 (83)	16 (80)	
NYHA IV, *n* (%)	2 (7)	2 (10)	
Diabetes, *n* (%)	25 (83)	19 (95)	0.14
Hypertension, *n* (%)	17 (89.5)	11 (100)	0.38
Previous myocardial infarction, *n* (%)	4 (13.3)	3 (15)	1.00
Previous PCI, *n* (%)	7 (23.3)	4 (20)	1.00
Previous CABG, *n* (%)	3 (10)	2 (10)	1.00
Previous stroke, *n* (%)	3 (10)	3 (15)	0.67
COPD, *n* (%)	5 (16.7)	2 (10)	0.69
AF baseline, *n* (%)	29 (96.7)	16 (80)	0.14
AF 1‐year follow‐up, *n* (%)	9 (69.2)	6 (88.2)	0.36
BNP baseline (pg/mL)	404.5 (184 to 805)	392.5 (227.5 to 769.8)	0.11
BNP discharge (pg/mL)	319 (197.8 to 526)	560 (279.3 to 929)	**0.07**
BNP 1‐month follow‐up (pg/mL)	287.5 (191.3 to 386.3)	506.5 (223.3 to 935.5)	**0.06**
BNP 1‐year follow‐up (pg/mL)	431.5 (188 to 576.3)	317 (257.8 to 691.3)	0.33
Medication, *n* (%)			
Loop diuretics	27 (93.1)	18 (90)	1.00
ACEI or ARB	21 (72.4)	16 (80)	0.74
Beta‐blockers	21 (72.4)	18 (90)	0.17
Aldosterone antagonists	11 (37.9)	4 (20)	0.22
Baseline echocardiographic data			
LVEF biplane (%)	54.9 ± 7.4	56.7 ± 7.5	0.41
LVEDVi (Simpson, m/m^2^)	56.1 ± 20.5	56.8 ± 22.2	0.90
LVESVi (Simpson, mL/m^2^)	25.2 ± 10.9	24.7 ± 10.5	0.88
LAESV (mL)	145.3 ± 51.4	120.5 ± 57.0	0.11
LAEDV (mL)	110.9 ± 44.0	97.1 ± 48.4	0.30
LAEF biplane (%)	0.24 ± 0.11	0.20 ± 0.11	0.15
LAV AL (mL)	169.3 ± 55.0	140.7 ± 64.3	0.12
LAVI AL (mL/m^2^)	99.9 ± 42.5	92.4 ± 36.1	0.54
LA_A_ (cm^2^)	34.8 ± 7.4	31.2 ± 8.3	**0.09**
LA_L_ (mm)	7.2 ± 0.7	7.0 ± 0.9	0.26
MR, *n* (%)			
N/A	0 (0)	0 (0)	0.82
1+	0 (0)	0 (0)	
2+	5 (17)	4 (19)	
3+	25 (83)	17 (81)	
MR VC (biplane, cm)	0.8 ± 0.3	0.8 ± 0.2	0.38
EROA (cm^2^)	0.19 ± 0.9	0.26 ± 0.17	0.15
MR PISA (cm)	0.7 ± 0.2	0.7 ± 0.2	0.94
MR Reg Volume (PISA, mL)	34.3 ± 17.1	42.4 ± 25.5	0.32
MVA ALPM (mm)	39.0 ± 8.2	40.2 ± 4.8	0.59
MVA AP (mm)	41.2 ± 3.8	41.5 ± 4.3	0.81
Tricuspid regurgitation grade, *n* (%)			0.88
N/A	2 (7)	1 (5)	
1+	5 (17)	5 (24)	
2+	7 (24)	6 (29)	
3+	15 (52)	9 (43)	
TVmax (m/s)	3.0 ± 0.5	3.2 ± 0.5	0.35
E (cm/s)	99.4 ± 31.7	107.1 ± 14.3	0.45
E′ med (cm/s)	7.6 ± 2.3	7.6 ± 1.3	0.99
E′ lat (cm/s)	10.2 ± 3.2	11.1 ± 2.4	0.47
E/E′ average	12.6 ± 6.0	11.1 ± 2.3	0.44
Peri‐procedural data			
Pre‐LA a (mmHg)	18.9 ± 7.0	16.8 ± 4.4	0.44
Pre‐LA v (mmHg)	24.6 ± 8.8	30.1 ± 11.1	0.14
Pre‐LA m (mmHg)	16.6 ± 4.9	17.3 ± 3.6	0.64
Pre‐MV MPG (mmHg)	1.6 ± 0.8	1.9 ± 1.0	0.22
Post‐LA a (mmHg)	16.6 ± 6.2	13.8 ± 4.2	0.22
Post‐LA v (mmHg)	18.8 ± 10.2	20.2 ± 7.6	0.66
Post‐LA m (mmHg)	13.1 ± 4.0	13.0 ± 3.1	0.93
Post‐MV MPG (mmHg)	2.2 ± 0.8	2.8 ± 1.1	**0.02**
Post‐MR grade, *n* (%)			0.78
N/A	6 (20)	4 (19)	
1+	21 (70)	16 (43)	
2+	3 (10)	1 (5)	
3+	0 (0)	0 (0)	
Device success = 1	29 (96.7)	20 (95.2)	1.00
Echocardiographic characteristics at 1 year			
LAESV (mL)	100.7 ± 39.2	122.7 ± 80.3	0.20
LAEDV (mL)	81.4 ± 39.1	97.9 ± 67.7	0.28
LA‐EF biplane	0.22 ± 0.11	0.21 ± 0.95	0.69
LAV AL (mL)	126.71 ± 25.28	128.83 ± 28.52	0.59
LAVI AL (mL)	80.46 ± 19.51	82.21 ± 15.43	0.21
LA_A_ (cm^2^)	27.4 ± 7.4	31.2 ± 10.9	0.14
LA_L_ (mm)	6.70 ± 0.80	6.87 ± 0.84	0.30
Mitral regurgitation grade, *n* (%)			0.31
0	10 (33)	3 (14)	
1	18 (60)	15 (71)	
2	2 (7)	2 (10)	
3	0 (0)	1 (5)	
MV MPG	3.1 ± 1.3	3.6 ± 1.7	0.22
Tricuspid regurgitation grade, *n* (%)			0.50
0	2 (7)	0 (0)	
1	15 (52)	9 (45)	
2	7 (24)	5 (25)	
3	5 (17)	6 (30)	
TVmax (m/s)	2.9 ± 0.5	2.9 ± 0.4	0.95
E (cm/s)	113.4 ± 43.0	129.8 ± 45.4	0.35
E′ med (cm/s)	5.6 ± 1.5	5.2 ± 1.5	0.57
E′ lat (cm/s)	8.6 ± 2.6	8.4 ± 2.8	0.88
E/E′	15.7 ± 6.9	19.3 ± 7.3	0.21

Data are presented as *n* (%), mean (SD) or median (interquartile range).

a, a‐wave; ACEI, angiotensin‐converting enzyme inhibitor; ALPM, anterolateral to posteromedial; AP, anteroposterior; ARB, angiotensin receptor blocker; BNP, brain natriuretic peptide; BSA, body surface area; CABG, coronary artery bypass surgery; COPD, chronic obstructive pulmonary disease; E, early mitral inflow velocity; E/E′, ratio between early mitral inflow velocity and mitral annular early diastolic velocity; E′, mitral annular early diastolic velocity; LA_A_, left atrial area; LAEDV, left atrial end‐diastolic volume; LAEF, left atrial ejection fraction; LAESV, left atrial end‐systolic volume; LA_L_, left atrial length; LVEDVi, left ventricular end‐diastolic volume index; LVEF, left ventricular ejection fraction; LVESVi, left atrial end‐systolic volume index; m, mean pressure; MR, mitral regurgitation; MVA, mitral valve annulus; NYHA, New York Heart Association; PCI, percutaneous coronary intervention; PISA, proximal isovelocity surface area; v, v‐wave; VC, vena contracta.

## Discussion

The present analysis describes the characteristics of patients with ASMR and the outcomes after M‐TEER, and evaluates the therapy's effects on reverse remodelling of the LA. To our knowledge, this is the first study that reports the incidence of LARR at 1 year and its association with clinical, echocardiographic and peri‐procedural parameters in ASMR patients undergoing M‐TEER.

Although ASMR has been increasingly recognized, there is still much controversy regarding the optimal treatment for this entity due to a lack of evidence. M‐TEER is an emerging treatment option for SMR, whereas there are still no distinct recommendations for percutaneous interventions.^6^ SMR including ASMR patients display a heterogeneous patient collective including a relevant proportion at elevated risk for cardiac surgery, as reflected by the patient cohort of the present study. The prevalence of ASMR of 17.3% in the present study was similar to that reported in some consecutive series of patients undergoing M‐TEER,^9^, ^10^ however, higher than in other registries, which may be due to the discrepancy in ASMR definition.^7^, ^8^, ^11^, ^12^, ^26^ Nevertheless, this confirms that this population represents a small, but relevant fraction of SMR patients. The baseline characteristics of patients in our study were comparable to those observed in other registries investigating the interventional therapy for MR.^27^ As described by most registries including our analysis, ASMR patients are predominantly women, and the prevalence of AFib and hypertension are higher than in most other MR‐cohorts. In our cohort, 71% were female, and 89% of patients had pre‐existing AFib, while 84% were suffering from hypertension.

Evidence on effects of M‐TEER in patients with ASMR is still very scarce in comparison to ventricular SMR and prospective study results are lacking, since both published randomized‐controlled trials for the treatment of SMR by M‐TEER—COAPT and MITRA‐FR—had excluded patients with LV preserved systolic function.^28^, ^29^ The third randomized clinical trial on SMR patients, RESHAPE‐HF2—which was recently published, also included only patients with an LV‐EF up to 50%.^30^ However, according to previous reports on ASMR, MR is related to pathological LA remodelling leading to mitral annular dilatation and flattened leaflets, in contrast to ventricular SMR resulting from LV remodelling and dysfunction.^7^, ^8^, ^9^, ^10^, ^11^, ^12^ As the procedural results of M‐TEER depend on the underlying MV anatomy, the feasibility and the clinical impact of M‐TEER on ASMR needs further evidence. In the current study, the technical success was 100%, and an ‘acceptable’ procedural success at discharge was achieved in 92% of patients, with 100% of patients having an MR ≤ 2. Reports from other cohorts of ASMR patients and those with ventricular SMR undergoing M‐TEER showed comparable rates of procedural success over 90% at discharge, showing that M‐TEER exhibits similar clinical efficacy.^8^, ^9^, ^11^, ^26^, ^31^ Moreover, Doldi et al. showed in a large multicentric study that ASMR, ventricular SMR and non‐ASMR patients (defined as having normal LV‐EF, non‐Carpentier type I, or presence of regional wall motion abnormalities) have similar procedural and survival outcomes after M‐TEER, with a lower rate of post‐procedural MR ≤ 2 in ASMR patients. Therefore, the mechanism of ASMR does not appear to negatively influence post‐procedural outcomes. In our study, survival rate was 95% at 1YFUP, with a significant improvement in NYHA functional class that persisted at follow‐up. These findings suggest that M‐TEER is a feasible, safe and effective therapeutic option for patients with clinically significant ASMR.

Patients with ASMR have higher rates of TR with larger left and right atria, compared with those with ventricular SMR which might suggest more advanced cardiac remodelling.^9^, ^11^, ^12^, ^26^, ^31^ In our cohort, 72% patients had moderate to severe TR at baseline, similar to previous reports.^11^, ^12^, ^26^, ^31^ The rate of patients with severe TR at baseline was 54%, of which 26% underwent TEER for TR either concomitantly or post‐M‐TEER.

Our study gives further directions in the treatment of ASMR patients. ASMR typically occurs in the context of severe LA enlargement and subsequent dilatation of the MV annulus, commonly in two important scenarios, namely, in the presence of AFib and/or HFpEF.^8^, ^9^, ^10^ Similar to data reported by Doldi et al.,^11^ in the present study the majority of patients had a history of AFib, confirming the strong association with ASMR in this subgroup. Given the key drivers of LA remodelling, the control of traditional risk factors including arterial hypertension and successful cardioversion or ablation of AFib have been shown to promote reverse remodelling with improvement in LA function and reduction of LA volumes at early stages.^3^, ^24^, ^32^ In our study, we observed a significant reduction in LA volumes at 1YFUP after M‐TEER, with an improvement of LA‐EF in 43% of patients, showing that M‐TEER might have the potential to address LA remodelling also at later stages, when volume overload due to significant MR has already been fuelling the vicious cycle. Further, LARR defined in the present study as LAESV decrease ≥ 15% at 1 year, was achieved in 59% of patients who underwent successful M‐TEER. Post‐procedural MR severity did not differ significantly between both groups; however, patients in LARR had lower post‐procedural mitral valve MPG and peri‐procedural BNP values. A multivariate logistic regression analysis identified pre‐procedural MPG and BNP at discharge as independent predictors for the occurrence of LARR at 1 year. Nonetheless, this study is limited by the nature of an observational study and the small sample size; therefore, meaningful conclusions related to predictors of LA positive remodelling cannot be drawn without further research on larger cohorts. Future studies will be required to reveal the best timing for MV interventions before irreversible LA remodelling with fibrosis has occurred.

### Study limitations

The retrospective design of this study, along with the small sample size makes hidden confounders likely. Therefore, the data should be interpreted as ‘hypothesis generating’. Due to the non‐randomized and monocentric study design, a selection bias is possible. Yet the inclusion of consecutive patients in an ‘all‐comers’ fashion somewhat compensates for this. Another aspect of the small sample size is the limitation to detect small statistically significant differences mostly between LARR vs. NLARR groups; therefore, the findings need to be validated in larger future studies.

Echocardiographic data were not evaluated by an independent core lab. However, data were assessed by an experienced physician in accordance with society recommendations. Since there is no general consensus, the definitions for ASMR and LA reverse remodelling vary between studies, which may lead to differences in the prevalence of specific characteristics and outcomes of the patients. Additionally, as the imaging protocol was not specifically optimized for atrial strain assessment, high‐resolution and left atrium‐focused images were not consistently acquired. As a result, strain measurements were deemed unreliable and were therefore not included in the analysis.

Future studies are needed to evaluate the prognosis and the treatment effectiveness of M‐TEER, which patients benefit more, and are more likely to achieve a reverse remodelling of the LA.

## Conclusions

Transcatheter mitral valve repair by edge‐to‐edge therapy in symptomatic patients with atrial secondary mitral regurgitation is safe, effective, and might have the potential to induce left atrial reverse remodelling in selected patients. Future studies are needed to determine the optimal patient characteristics and timing for intervention in order to achieve an optimized result in this patient population.

## Funding

None declared.
